# Relationship between adult attachment and cognitive emotional regulation style in women and men

**DOI:** 10.1038/s41598-023-35250-0

**Published:** 2023-05-19

**Authors:** Begoña Delgado, Pedro J. Amor, Francisco J. Domínguez-Sánchez, Francisco P. Holgado-Tello

**Affiliations:** Departamento de Psicología de la Personalidad, Evaluación y Tratamiento Psicológicos, Facultad de Psicología, Universidad Nacional de Educación a Distancia, C/ Juan del Rosal, 10, 28040 Madrid, Spain

**Keywords:** Psychology, Human behaviour

## Abstract

Cognitive emotion regulation (CER) strategies are useful in evaluating the risk of developing emotional disorders and that they may define subjects’ styles. This study aims to explore the extent to which specific styles of CER strategies relate to the anxious and avoidant attachment dimensions in adults and whether such relationships operate similarly for women and men. Two hundred and fifteen adults (between 22 and 67 years old) completed the Spanish versions of the Cognitive Emotion Regulation Questionnaire and the Experiences in Close Relationships instrument. Cluster analysis, ANOVA and Student's t-test were used. Our results show that women and men can be successfully classified into two CER clusters (Protective and Vulnerable), distinguished by the higher use in the protective cluster of the CER strategies considered most adaptive and complex (Acceptance, Positive Refocusing, Refocus on Planning, Positive Reappraisal, and Putting into Perspective). However, only in women were the anxious and avoidant attachment dimensions significantly associated with CER style. In conclusion, from a clinical and interpersonal perspective, it is interesting to be able to predict the belonging to a Protective or Vulnerable coping style by analysing the CER strategies and to know their relationship with the adult affective system.

## Introduction

Emotion regulation refers to the set of competences that allow one to supervise, evaluate, and modify the processes that are implied in the origin of emotion, thereby modulating one’s emotional manifestations^1^. Due to its importance, emotion regulation has been addressed by different lines of research from a range of perspectives, including the biological, psychological, and socio-cultural. In this broad view, cognitive emotion regulation strategies are highlighted as a factor relevant to understanding our way of dealing with emotional threats. Garnefski et al.^2^ developed the Cognitive Emotion Regulation Questionnaire (CERQ) to assess the thoughts most employed to handle challenging emotions or feelings, such as *Self-Blame, Acceptance, Rumination, Positive Refocusing, Refocus on Planning, Positive Reappraisal, Putting into Perspective, Catastrophizing,* and *Blaming Others*. Subsequent research has confirmed the presence of these nine primary strategies across different cultures and ages^3–10^. Research on cognitive emotion regulation strategies has revealed that, on the one hand, strategies usually referred to as “less adaptive” or “maladaptive,” such as *Rumination*, *Catastrophizing*, *Self-Blame,* and *Blaming Others*, are directly related to symptoms of depression and anxiety^3–6^. On the other hand, the so-called “adaptive strategies,” such as *Positive Reappraisal*, *Putting into Perspective*, and *Acceptance*, are inversely related to such symptoms (e.g.,^7–10^).

However, the functional relationship that defines maladaptive strategies as risk factors and adaptive strategies as prevention factors is not always applicable. It varies with such factors as the condition of the sample, e.g., clinical or community-dwelling, its age, and its cultural composition^11–13^. At the same time, various works have proposed that the adaptive or maladaptive nature of the strategies and, therefore, their protective or risk-enhancing function, also depends on various contextual factors^14–17^. The research available in this regard is still very scarce; so far, for example, the analysis has focused on contextual factors such as the controllability of the stressor^18^, the type or intensity of the emotion, and the social or achievement circumstances in which the strategies must be implemented^19^. In general, the results obtained describe a dynamic process in which contextual factors mediate the selection of whatever strategies may be the most effective in the specific circumstances that the person must face. These works, essentially, focus on the study of the cognitive and emotional effects associated with the regulation of the emotional response. However, most affective transactions take place in social settings. In fact, interpersonal processes (e.g., in a family, social, or couple setting) are a contextual factor that is especially relevant in terms of the emotions we experience and the way we regulate them^20^. Moreover, the research carried out from this interpersonal perspective is of relevance insofar as it provides information not only on the intrapsychic processes that underlie the regulation of emotion, but also on the effects of that regulation on different areas of a person’s life (e.g., health, affiliative tendencies, relationships, and conflict management)^20,21^. In relation to this, it seems evident that the styles of affective bonding that we deploy in interpersonal relationships could affect the processes that give rise to emotion and its regulation. The theory of attachment formulated by Bowlby^22^ looks at the bond of attachment from the perspective of how a baby–adult interaction system operates to keep the child close to the adult and to protect him or her from threats. According to this theory, as the child grows, the experiences of caring begin to be represented symbolically in an internal working model that gathers the essential aspects of the self and the other in an attachment relationship. However, as pointed out by Bretherton and Munholland^23^, in Bowlby’s theory^22^, internal working models should not be regarded as dispositions or temperament traits, since they are updated as the child develops. Numerous studies highlight the stability of an early attachment style during childhood and adolescence, especially the secure style^24^. The principles of attachment theory have been applied to adult relationships, revealing the presence of parallel styles of emotional relationships and threat coping. Nevertheless, correspondences between early attachment representations and romantic styles need to be addressed carefully. Measures of adult attachment, as the state of mind with respect to attachment, assessed by the AAI (Adult Attachment Interview) have shown a wide association with early attachment representations^24^. However, despite their conceptual analogies, mainstream research reports a weak relationship between the affective style in current romantic relationships and early attachment representations^24,25^. It has been speculated that the patterns of affective regulation observed in adult relations could be more strongly affected by current interpersonal exchanges. Therefore, the affective style displayed in adult relationships might be affected by factors that go beyond early attachment representations (such as previous experiences in romantic relationships, maturative changes, or significant life situations). Furthermore, previous research has proven that the adult affective style influences different areas of a person’s life, emerging as a relevant dimension for our understanding of adults’ cognitive emotion regulation styles. In general, anxiously attached adult individuals have been described as having a higher sensitivity for detecting threats and a bias towards a negative and exaggerated valuation of such threats. Alternatively, avoidantly attached adult individuals are defined by their attempts to render the system of attachment through methods such as emphasizing self-sufficiency, avoiding emotional closeness, denying their attachment needs, and maximizing their physical and emotional distance from others^26,27^. Predictably, insecure adult attachment dimensions have been related to symptoms of depression and anxiety or psychopathic traits^28–31^.

As far as we know, the relationship between an individual’s adult romantic style and their profile of cognitive emotion regulation remains unexplored, even when it is predictable that the two constructs may be interrelated. To fill this gap, in this work we explore the relationship between adult cognitive emotion regulation style and the avoidant and anxious adult attachment dimensions in the context of a romantic relationship, examining whether such a connection works in a similar way for men and women. We hope that this work will improve our knowledge about the cognitive style of emotional regulation in women and men and about how these styles relate to the affective bonds that both maintain with their partners.

## Method

### Participants

A total of 215 people participated in the study, ranging in age from 22 to 67 years old (*M* = 41.03 years old, *SD* = 10.49). Of the participants, 30.2% were men (*M* = 42.20 years old, *SD* = 10.29), while 69.8% were women (*M* = 40.53 years old, *SD* = 10.61). Regarding academic level, most of the sample held a university degree (87%), and the rest of the participants had professional training (6.5%) or had graduated from secondary school (4.7%) or primary school (1.9%). Employment status was also taken into consideration: 48.4% had a permanent job, 14% had a temporary job, 2.3% were retired, and the rest of the participants either did not work (13.5%) or were focusing exclusively on their studies (21.9%). Participation in this research was voluntary, and there were no economic or academic rewards.

### Instruments

*The Cognitive Emotion Regulation Questionnaire*^2^, Spanish version (CERQ-S)^32^ consists of 36 items, four for each of the nine cognitive emotion regulation strategies it measures: *Self-Blame* (i.e., thinking that one is responsible for what happened), *Acceptance* (i.e., accepting what has happened and resigning oneself to it), *Rumination* (i.e., reflecting on one’s feelings and thoughts associated with what happened), *Positive Refocusing* (i.e., thinking about enjoyable experiences instead of about the stressful event), *Refocus on Planning* (i.e., concentrating on the measures to adopt in response to the event), *Positive Reappraisal* (i.e., considering the positive aspects of what happened), *Putting into Perspective* (i.e., reducing the relevance of the event), *Catastrophizing* (i.e., having thoughts that intensify the negative side of what happened), and *Blaming Others* (i.e., having thoughts that shift the blame for what happened onto others). In turn, these nine scales can be grouped into two more general categories, namely adaptive or appropriate strategies (*Acceptance*, *Positive Refocusing*, *Refocus on Planning*, *Positive Reappraisal*, and *Putting into Perspective*) and maladaptive or inappropriate strategies (*Self-Blame*, *Rumination*, *Catastrophizing*, and *Blaming Others*)^2,32,33^. These two categories (more adaptive strategies and less adaptive strategies) will be the ones used in this research and are the result of the sum of the corresponding CERQ first-order dimensions. The CERQ has good psychometric properties, both in its original version^2^ and in the versions adapted to other languages, in which the structure of the original nine-factor model has been confirmed^6,32–35^. In the sample used in the original study^2,36^, the internal consistency (alpha) of the scales ranged from 0.75 (Self-Blame) to 0.86 (Refocus on Planning). In our study the alpha values ranged from 0.70 (Acceptance) to 0.89 (Positive Reappraisal), with coefficient alphas of 0.89 and 0.82 for the more and less adaptive strategies, respectively.

*Experiences in Close Relationships*^37^ Spanish version (ECR-S)^38^ is a 32-item instrument containing a 7-point (1 = “strongly disagree” and 7 = “strongly agree”) Likert-type answer scale that has been validated within the adult Spanish population. It enables the evaluation of the original ECR’s orthogonal romantic attachment dimensions of anxiety and avoidance. In the ECR-S, 17 items measure anxiety about relationships (for instance, “*I resent it when my partner spends time away from me*”) and 15 items measure avoidance of intimacy (e.g., “*I am nervous when partners get too close to me*”). The items appear in the same order as in the English-language ECR. The ECR has good psychometric properties, both in its original version^37^ and in the Spanish version^38^. According to its authors, the ECR-S presents indexes of satisfactory internal consistency (Cronbach’s alpha) of 0.87 for the *Avoidance* dimension and 0.85 for the *Anxiety* dimension. In our study, the alpha values ranged from 0.92 for *Avoidance* to 0.88 for *Anxiety*.

### Procedure

This cross-sectional, relational, and descriptive research study was carried out between January 2020 and December 2022. The sample was obtained through a form that was uploaded on an institutional open web page of the Universidad Nacional de Educación a Distancia (UNED; https://www.uned.es). They were informed that their participation, which was voluntary and anonymous, would comprise completing a series of sociodemographic data and two questionnaires (CERQ-S and ECR-S). All participants signed the informed consent through the Internet form (by checking the corresponding checkbox). This study was approved by the ethics committee of the Universidad Nacional de Educación a Distancia (protocol number 25-PSI-2022) and was conducted in accordance with the Declaration of Helsinki (World Medical Association)^39^.

Not being 18 years of age at the time of the study and not having signed the informed consent were considered exclusion criteria for the study.

Although the total sample consisted of 215 participants, five cases were eliminated from the analyses because three participants did not indicate their gender and two left unanswered more than four of the instrument’s items measuring attachment. In the remaining cases, missing values for an item were replaced by the mean value of the item. The missing values, distributed randomly among the different items from these scales, represent 0.32% of the data from the Cognitive Emotion Regulation Questionnaire and 0.36% from the Experiences in Close Relationships instrument.

### Data analysis

The statistical analyses employed were as follows: Descriptive analysis and Pearson's correlations between the first and second order dimensions of the CERQ and the attachment dimensions (avoidance and anxiety). Cluster analysis (K-means) was used to obtain groups of subjects who were similar in their cognitive emotion regulation style (in all cases, the criterion of K = 2 was used, which is the minimum value that allows capturing differences between participants). This analysis was used for the total sample and later also separately for the all-male and all-female subsamples. The clustering process used the scores from the subjects in the second-order factor dimensions of the CERQ-S and used One-Way ANOVA to examine the relevance of the variables in the process of conglomeration. Student’s *t*-test and Hedges’ *g* (95% interval confidence) were used to analyse the possible statistical differences and the effect size (ES) between the two coping styles identified in each case (total sample and separate sub-samples of men and women) in the nine first-order dimensions of the CERQ-S and in the two dimensions of adult attachment. IBM SPSS 27 was used for the statistical analysis.

## Results

### Descriptive analysis

Descriptive analysis and correlation matrix were calculated between the first and second-order dimensions of the CERQ and avoidance and anxiety attachment dimensions (Table 1).

**Table 1:** Descriptive Statistics and Correlations Between Cognitive Emotional Regulation Questionnaire and Attachment Dimensions.

Variable	*M*	*DT*	1	2	3	4	5	6	7	8	9	10	11	12
1. More adaptive	70.20	12.22	–											
2. Less adaptive	38.65	7.89	.02	–										
3. Self-blame	10.42	2.63	.01	.58**	–									
4. Acceptance	13.60	3.03	.50**	.29**	.20**	–								
5. Rumination	13.11	3.42	.09	.79**	.34**	.29**	–							
6. Positive refocusing	10.96	3.77	.72**	−.04	−.13	.18**	−.04	–						
7. Refocus on planning	16.48	2.95	.75**	.02	.01	.26**	.14*	.39**	–					
8. Positive reappraisal	15.77	3.82	.82**	−.17*	−.01	.21**	−.07	.46**	.66**	–				
9. Putting into perspective	13.39	3.47	.76**	.01	.03	.24**	.06	.44**	.42**	.56**	–			
10. Catastrophizing	7.00	2.59	−.15*	.78**	.33**	.14*	.43**	−.04	−.18**	−.30**	−.12	–		
11. Blaming others	8.12	2.69	.07	.61**	−.02	.17*	.30**	.09	.05	−.10	.05	.44**	–	
12. Avoidance	42.25	19.26	−.22**	.26**	.08	.09	.12	−.09	−.20**	−.27**	−.27**	.35**	.20**	–
13. Anxiety	44.56	16.32	−.28**	.34**	.11	.02	.24**	−.22**	−.26**	−.34**	−.17*	.37**	.23**	.21**

* *p* < .05, * *p* < .01.

### Cluster analysis according to cognitive emotional coping type

**Figure 1:**
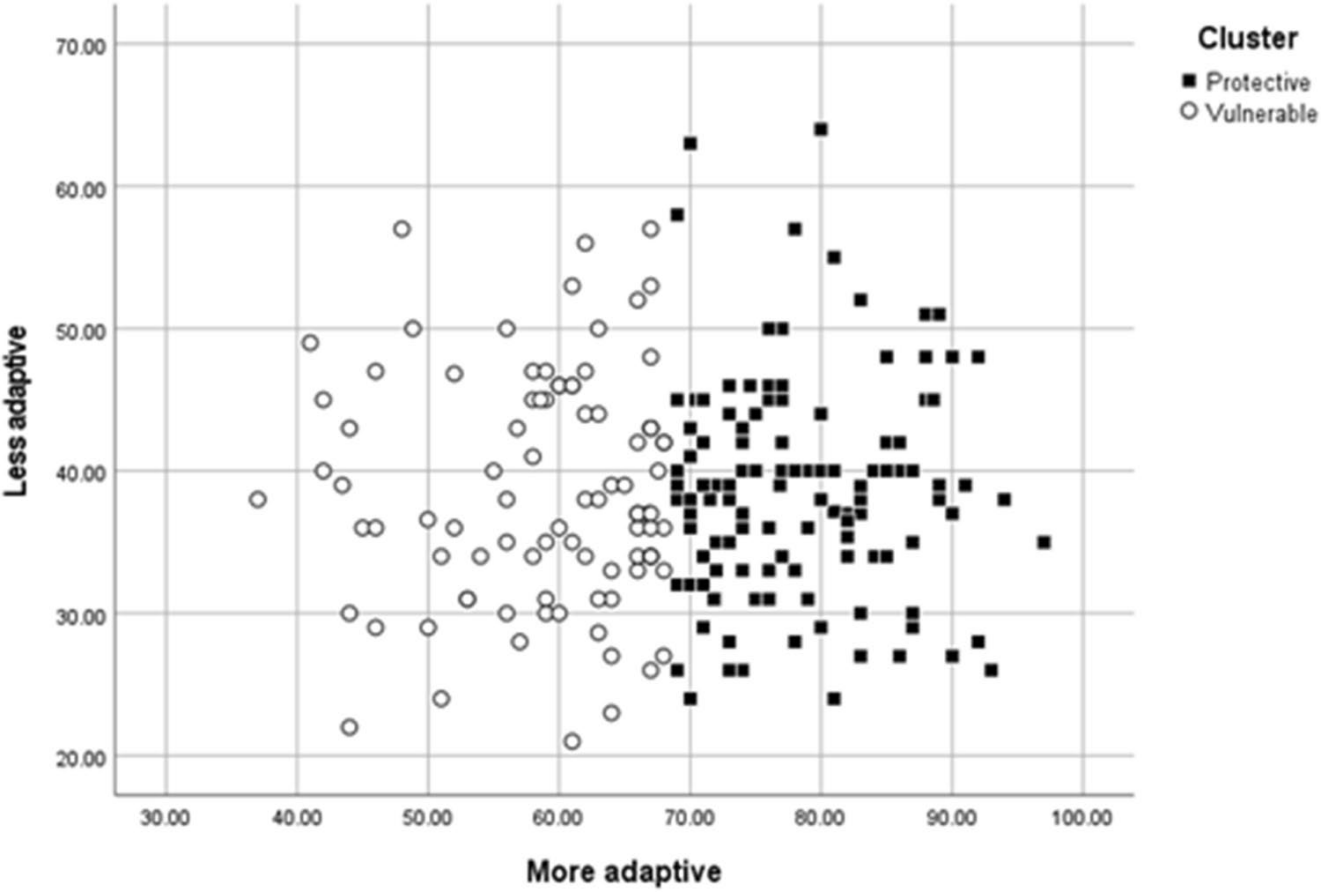
Style 1 (Protective) and Style 2 (Vulnerable) Formed According to the Mean Scores from the More Adaptive and Less Adaptive Strategies.

To construct the clusters, the participants’ scores on the second-order factors on the CERQ (*More* vs. *Less* adaptive strategies) were employed as variables. Two groups were obtained (Table 2 shows the results of the clusterization process and the centres of the final clusters). The first cluster consisted of 124 participants, who were grouped together according to their ability to use a higher amount of the more adaptive cognitive emotional coping strategies (CECS): *Acceptance*, *Positive Refocusing*, *Refocus on Planning*, *Positive Reappraisal*, and *Putting into Perspective* (*Protective* style). The second cluster consisted of 91 participants, who were grouped together based on their minor tendency to use the more adaptive CECS, resulting in a more equitable use of the more and the less adaptive cognitive emotional regulation strategies (*Vulnerable* style). In both groups, the use of the less adaptive strategies was similar (Fig. 1). In summary, in one group the use of adaptive strategies is clearly higher than that of non-adaptive ones (*protective* group); while in another group, clearly differentiated, the use of adaptive strategies is simply superior to that of non-adaptive strategies (*vulnerable* group).

**Table 2:** ANOVA of the Variables in the Clustering Process in the Total Sample.

Variable	Cluster 1		Error			
	Quadratic mean	*df*	Quadratic mean	*df*	*F*	*p*
More adaptive	20,105.23	1	55.22	213	364.09	< .001
Less adaptive	0.748	1	62.45	213	0.01	.913

Cluster centres						
	Style 1 (*Protective*) (*n*_*1*_ = 124)	Style 2 (*Vulnerable*) (*n*_*2*_ = 91)				
More adaptive	78.44	58.87				
Less adaptive	38.64	38.52				

K-means cluster solution. The lower part of this table indicates the centres of the final clusters.

Subsequently, the two groups were compared in the nine first-order dimensions of the CERQ-S. Significant statistical differences were found in five of the nine dimensions of the CERQ-S considered more adaptive. The participants from group 1 (*Protective* style) scored significantly higher than the participants from group 2 (*Vulnerable* style) in the dimensions of *Positive Refocusing*, *Refocus on Planning*, *Positive Reappraisal*, *Putting into Perspective* (large ES), and *Acceptance* (medium ES). In contrast, no statistically significant differences were found between the two groups in the use of *Self-Blame*, *Rumination*, *Catastrophizing*, or *Blaming Others*. The results indicate that the most relevant strategies for differentiating between both coping styles are those described above as the “more adaptive” strategies (see Table 3).

**Table 3:** Means, Standard Deviations, T-test, and Effect Size for Comparisons Between the Two Styles in the CERQ-S and Attachment Dimensions.

Variable	Style 1 (*n*_*1*_ = 122)		Style 2 (*n*_*2*_ = 88)		*t*	*df*	*p*	Effect size	95% CI	
	*M*	*SD*	*M*	*SD*				*g*	*LL*	*UL*
Self-blame	10.46	2.63	10.43	2.61	0.07	208	.941	0.01	− 0.26	0.28
Acceptance	14.48	2.91	12.34	2.76	5.38	208	< .001	0.75	0.47	1.03
Rumination	13.23	3.38	13.00	3.48	0.49	208	.625	0.07	− 0.20	0.34
Positive refocusing^a^	12.80	3.33	8.43	2.75	10.37	204.17	< .001	1.40	1.10	1.70
Refocus on planning^a^	17.88	2.09	14.66	2.88	8.92	149.77	< .001	1.31	1.01	1.61
Positive reappraisal^a^	17.99	2.01	12.76	3.67	12.12	124.68	< .001	1.84	1.52	2.17
Putting into perspective	15.28	2.64	10.78	2.69	12.08	208	< .001	1.68	1.36	2.00
Catastrophizing	6.88	2.67	7.25	2.49	− 1.02	208	.308	− 0.14	− 0.42	0.13
Blaming others	8.14	2.54	8.16	2.92	− 0.04	208	.965	− 0.01	− 0.28	0.27
Avoidance^a^	38.81	17.52	46.48	20.80	− 2.81	167.28	.005	− 0.40	− 0.68	− 0.13
Anxiety	41.05	15.15	49.16	16.84	− 3.65	208	< .001	− 0.51	− 0.79	− 0.23

“Style 1” Protective, “Style 2” *Vulnerable*. “*g”* Hedges’ *g,* “95% CI” Confidence Interval (95%): *g* = .20 (Small); .50 (Medium); .80 (Large). ^a^ Welch test is reported because Levene’s test indicated that the homogeneity of variances assumption was not met for this variable.

### Analysis of attachment factors according to coping style

Once the two types of participants were identified according to their CECS style, their mean scores on the two dimensions of adult attachment evaluated via the ERC-S were compared. Statistically significant differences were found in both dimensions of attachment (Table 3). The participants from the *protective* style, characterized by the more frequent use of the more adaptive CECS, were shown to have lower scores on avoidance (small ES) and anxiety (medium ES) attachment dimensions.

### Cluster analysis according to cognitive emotional coping type by gender

The cluster analysis was repeated separately for the all-male and all-female samples. As in the previous case, two participants groups were obtained. Table 4 shows the results of the clusterization process and the centres of the final clusters for men and women. As in the previous analysis, the differences between the clusters are determined by the group of more adaptive strategies. In both samples, the people included in the first group (*Protective* style) are characterized by a higher score on the more adaptive CECS. In contrast, the use of the less adaptive strategies is similar for the four groups.

**Table 4:** ANOVA of the Variables in the Clustering Process in Men (and Women).

Variable	Cluster 1		Error		*F*	*p*
	Quadratic mean	*df*	Quadratic mean	*df*		
More adaptive	5,672.47 (13,697.25)	1	56.28 (55.97)	61 (145)	100.79 (244.70)	< .001 (< .001)
Less adaptive	41.42 (151.02)	1	68.03 (58.78)	61 (145)	0.61 (2.57)	.438 (.111)

Cluster centres						
	Style 1 (*Protective*) (*n*_*men*_ = 24; *n*_*women*_ = 87)	Style 2 (*Vulnerable*) (*n*_*men*_ = 39; *n*_*women*_ = 60)				
More adaptive	81.44 (78.70)	61.90 (59.06)				
Less adaptive	39.92 (37.87)	38.25 (39.93)				

Data for women in brackets.

**Figure 2:**
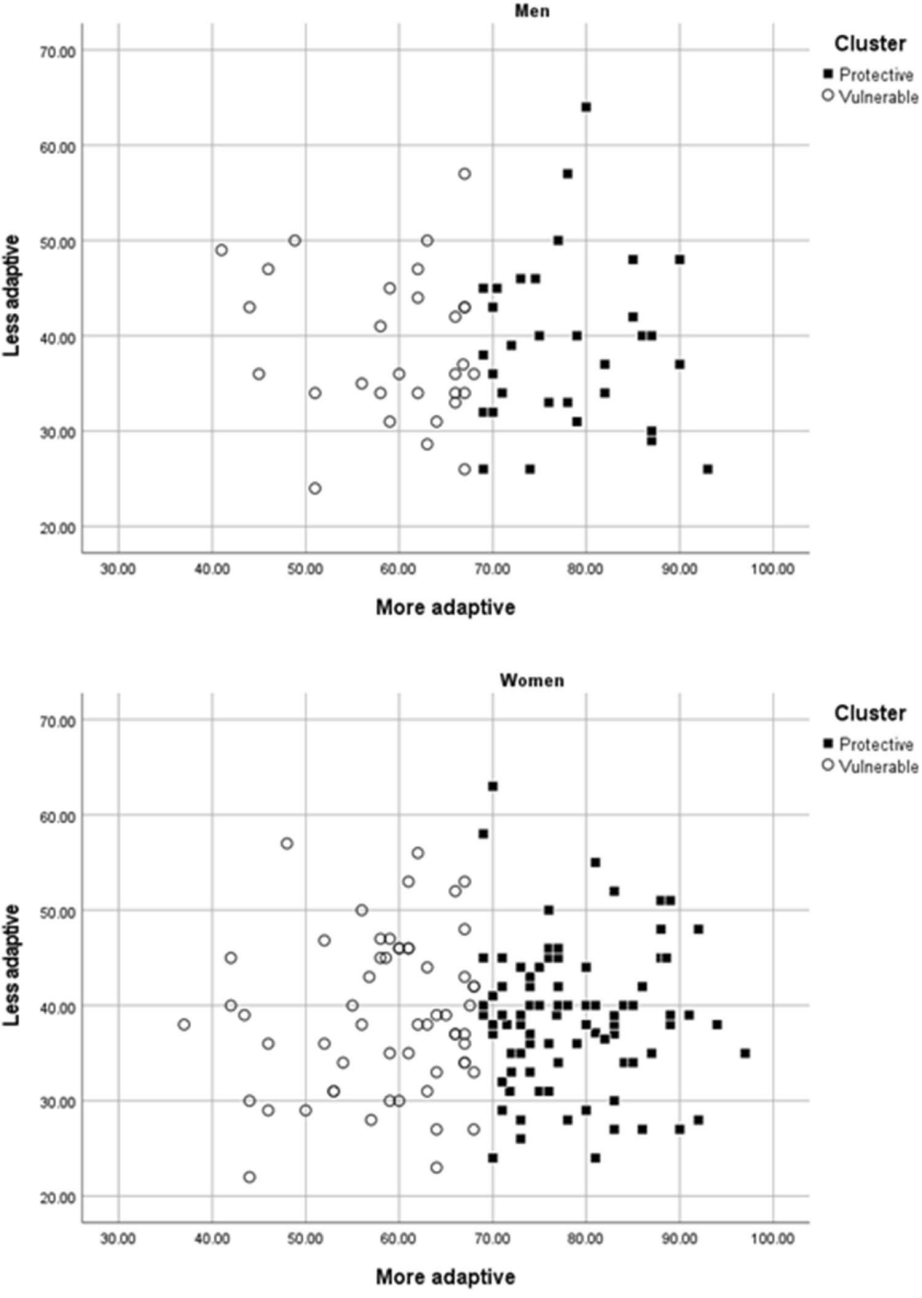
Style 1 (Protective) and Style 2 (Vulnerable), Formed According to the Mean Scores from the More Adaptive and Less Adaptive Strategies in Women and Men Separately.

The first group (*Protective* style) consisted of 24 men and 87 women, who were grouped together according to their ability to use a higher amount of the more adaptive CECS (*Acceptance*, *Positive Refocusing*, *Refocus on Planning*, *Positive Reappraisal*, and *Putting into Perspective*). The second group (*Vulnerable*) consisted of 39 men and 60 women. As can be seen in Fig. 2 (and similarly to Fig. 1), if a cut-off point were to be established to differentiate between both groups, it would be at around 70 points in the more adaptive strategies.

In comparing mean CECS scores according to group (*Protective* vs. *Vulnerable*), very similar results were obtained for men and women (see Table 5). Significant statistical differences were found in the five first-order dimensions of the CERQ-S considered to be more adaptive strategies. Specifically, the participants (men or women) from group 1 (*Protective* style) scored significantly higher than the participants from group 2 (*Vulnerable* style) in the dimensions of *Acceptance*, *Positive Refocusing*, *Refocus on Planning*, *Positive Reappraisal*, and *Putting into Perspective*, with large effect sizes, except for the group of women in the *Acceptance* dimension (medium ES). Regarding the strategies considered less adaptive, no statistically significant differences in *Self-Blame* or *Rumination* were found in either of the two groups. In contrast, different results were obtained in the group of men and women in the dimensions of *Catastrophizing* and *Blaming Others*. Specifically, on the one hand, women fitting group 2 (*Vulnerable* style) scored significantly higher than those aligning with group 1 (*Protective* style) on *Catastrophizing* (small ES); however, no statistically significant differences were found in men. On the other hand, the men from group 1 (*Protective* style) scored significantly higher than those from group 2 (*Vulnerable* style) on the strategy of *Blaming Others* (medium ES), in contrast to the women, among whom no statistically significant differences were found between the two styles.

**Table 5:** Means, Standard Deviations, T-test, and Effect Size for the Comparisons Between the Two Styles in the CERQ-S Dimensions in Men and Women Separately.

Variable	Gender	Style 1			Style 2			*t*	*df*	*p*	Effect size	95% CI	
		*n*	*M*	*SD*	*n*	*M*	*SD*				*g*	*LL*	*UL*
Self-blame	Men	24	9.79	2.34	39	10.69	2.68	−1.36	61	.179	−0.35	−0.85	0.16
	Women	87	10.53	2.54	60	10.43	2.81	0.22	145	.830	0.04	−0.29	0.36
Acceptance	Men	24	14.73	2.58	39	12.67	2.43	3.20	61	.002	0.82	0.29	1.34
	Women	87	14.56	3.01	60	12.32	2.98	4.46	145	< .001	0.75	0.41	1.08
Rumination	Men	24	13.21	3.19	39	12.22	3.60	1.10	61	.275	0.28	−0.22	0.79
	Women	87	13.25	3.37	60	13.52	3.44	−0.46	145	.646	−0.08	−0.40	0.25
Positive refocusing	Men	24	13.83	3.36	39	9.21	3.00	5.67	61	< .001	1.45	0.89	2.01
	Women^a^	87	12.83	3.33	60	8.27	2.50	9.46	143.88	< .001	1.50	1.13	1.87
Refocus on planning	Men^a^	24	18.63	1.50	39	14.77	2.90	6.94	59.61	< .001	1.55	0.97	2.11
	Women^a^	87	17.95	2.01	60	14.77	2.83	7.51	99.00	< .001	1.33	0.97	1.69
Positive reappraisal	Men^a^	24	18.63	1.61	39	13.69	4.01	6.84	54.37	< .001	1.47	0.90	2.03
	Women^a^	87	18.10	1.95	60	12.70	3.35	11.25	86.46	< .001	2.06	1.65	2.46
Putting into perspective	Men	24	15.63	2.41	39	11.56	2.89	5.76	61	< .001	1.48	0.90	2.04
	Women	87	15.26	2.79	60	11.00	2.89	8.96	145	< .001	1.50	1.12	1.86
Catastrophizing	Men	24	7.38	3.23	39	7.44	2.43	−0.09	61	.932	−0.02	−0.52	0.48
	Women	87	6.44	2.21	60	7.50	2.82	−2.55	145	.012	−0.43	−0.76	-0.09
Blaming others	Men	24	9.54	3.30	39	7.90	2.61	2.19	61	.032	0.56	0.05	1.07
	Women^a^	87	7.65	2.13	60	8.48	3.03	−1.84	98.09	.068	−0.33	−0.66	0.00
Avoidance	Men	24	41.42	20.92	39	46.88	22.02	−0.97	61	.334	−0.25	−0.75	0.26
	Women^a^	87	37.56	14.66	60	45.59	21.54	−2.52	96.00	.014	−0.45	−0.78	−0.12
Anxiety	Men	24	38.83	14.99	39	45.64	16.67	−1.63	61	.107	−0.42	−0.93	0.09
	Women	87	40.91	14.90	60	51.05	16.70	−3.86	145	< .001	−0.64	−0.98	-0.31

“Style 1” *Protective*, “Style 2” *Vulnerable*. “*g”* Hedges’ *g*, “95% CI” Confidence Interval (95%): *g* = .20 (Small); .50 (Medium); .80 (Large). ^a^ Welch test is reported because Levene’s test indicated that the homogeneity of variances assumption was not met for this variable.

### Analysis of attachment factors according to coping style by gender

This analysis was carried out on men and women separately. The two groups yielded different results in the attachment dimensions (see Table 5). In the group of men, no differences were found between the two cognitive emotional regulation styles in the *Anxious* and *Avoidant* attachment dimensions. In contrast, the women belonging to group 2 (*Vulnerable* style) scored significantly higher than those aligning with group 1 (*Protective* style) in the *Avoidant* (small ES) and *Anxious* (medium ES) attachment dimensions.

## Discussion

Cognitive strategies for emotional self-regulation play an important role in the way we deal with emotions and are of great relevance in clinical settings. Such strategies as *Rumination* or *Catastrophizing* are usually related to depression or anxiety symptoms^3–5,10^, while other strategies considered as “adaptive,” such as *Positive Refocusing* or *Putting into Perspective*, are inversely related to those same symptoms^7–10,14,40–42^. For this reason, results like those mentioned above have promoted the consideration of “multivariate patterns” of cognitive regulation strategies as an alternative to targeting isolated strategies of cognitive regulation^43^. Accordingly, our study approach focused not on isolated strategies, nor even on groupings of strategies, but on individual styles of the use of strategies, which emerges as an adequate method to produce a realistic image of how individuals use cognitive strategies for regulation.

The results of our work identified two cognitive styles of cognitive emotion regulation. Specifically, individuals with a noteworthy tendency to use the cognitive strategies usually related to higher mental health scores (*Acceptance*, *Positive Refocusing*, *Refocus on Planning*, *Positive Reappraisal*, and *Putting into Perspective*) fit a *Protective* style, which runs counter to a second, *Vulnerable* style composed primarily of individuals who make a significantly less noteworthy use of these same strategies. The statistical analyses also reveal that the use of the so-called adaptive strategies (*Acceptance*, *Positive Refocusing*, *Refocus on Planning*, *Positive Reappraisal*, and *Putting into Perspective*) is significant enough to distinguish individuals in terms of their style (see Fig. 1), suggesting that these cognitive emotion regulation strategies may be crucial for individuals to preserve psychological adjustment and avoid the risk of developing emotional disorders such as depression or anxiety. In terms of clinical implications, this result suggests the desirability of involving people in the use of "adaptive" and complex strategies, since these strategies are revealed as the discriminating factor between the two regulation styles observed. Interestingly, the current research agrees on highlighting the complexity and singularity of adaptive strategies, suggesting that *Positive Reappraisal*, *Positive Refocusing*, and *Putting into Perspective* may demand higher levels of attentional control abilities, which in turn implies that cognitive control deficits in working memory, interference control, or perseveration could lead to greater reliance on the cognitive strategies of *Self-Blame*, *Acceptance*, *Rumination*, and *Catastrophizing*^3,44,45^, which require less cognitive elaboration.

Traditionally, differences have been observed between men and women with respect to the greater use of one or another emotional regulation strategy^46,47^, noting, among other generalized effects, a greater presence of rumination or internalized coping styles in women^48^. These results are consistent with the significantly higher risk among women of developing a depression or anxiety disorder^46,49,50^.

Nevertheless, other research emphasizes that the nine cognitive emotional regulation strategies measured by the CERQ are gender-invariant, although women are more likely than men to use adaptive cognitive emotion regulation strategies^51^. In addition to these heterogenous results, some authors have pointed out that the relationship between the use of emotional regulation strategies and emotional disorders might not be the same for men and women^52,53^. In this puzzle of diverse results, in which not only cognitive or neurological but also cultural aspects have an influence, we find that from the point of view of the subjects, there are no differences in the cognitive regulation styles of men and women. In our study, male and female subjects showed the same two cognitive regulation styles, differentiated by the greater use of the most adaptive strategies. In addition, while the *Vulnerable* style described most of the men, in women the *Protective* style predominated – a result that is consistent with the evidence indicating that women tend to score higher in emotional intelligence^54–56^ and interpersonal competence^57–59^.

Although the cognitive coping groups showed identical clustering in men and women, a remarkable gender difference emerged. Specifically, men who fit the *Protective* style were significantly more likely to blame others than men allocated to the *Vulnerable* style (see Table 5). This issue, located in the cognitive coping groups of men, contrasts with the evidence that places this strategy among the least adaptive and relates it to the presence of psychopathy traits^51,60–63^, pointing again to the consideration already raised that the degree to which a regulatory strategy is adaptive depends not only on its nature but also on its flexible use and effective implementation by the individual^14–17^. Thus, while the rigid use of the *Blaming Others* strategy could generate maladaptive consequences for the individual, our study suggests that, in the broader context of a healthy and complex style of cognitive regulation, the use of this strategy could be “protective” for the user. *Blaming Others* is a strategy that involves externalizing responsibility, thus helping to avoid the emergence of negative emotions (e.g., guilt or shame)^64^ and to preserve the positive self-concept that the person has of himself^65,66^. In addition, *Blaming Others* is the strategy most linked to interpersonal relationships, as some authors have already pointed out^60^. This uniqueness puts us on the track of considering gender differences from the point of view of “the Other”. Recourse to *Blaming Others* might be more available to those people who have less of a tendency to put themselves in the *shoes of the other*, that is, to feel empathy. Empathy is a complex emotion that involves being able to share another person’s emotional state and mental perspective. This emotion traditionally shows higher levels in women, particularly in studies based on self-reported measures^67–69^. In turn, impassivity, a contrast factor to empathy defined as a lack of solidarity with and sensitivity to others, is a key factor in the research on gender differences^70^. Thus, although we cannot explain the reasons for the observed difference in the use of *Blaming Others* on the part of the men who fit the *Protective* style, based on previous research, we can hypothesize that a higher sensitivity to the perspectives of others and their feelings, usually found in women, may be underlying this gender singularity, which allows men to have one more strategy in their adaptive style of cognitive emotion regulation.

Gender differences were also found in the relationship between cognitive coping styles and adult attachment dimensions. As can be seen in Table 5, attachment dimensions were significantly related to the cognitive emotion regulation clusters of women, but they were not significant for men. Specifically, women who showed anxious or avoidant attachment in their relationships were more likely to be included in the *Vulnerable* cognitive emotion regulation group. That is, interpersonal attachment emerged as a more relevant factor in the cognitive emotional regulation processes of women than in those of men. The motives for this connection remain unclear, but we can hypothesize that this difference may be reinforced by the influence of cultural gender stereotypes. Parents socialize their sons and daughters according to culturally prescribed gender roles, which helps to explain why, as children become more aware of their images, sex role stereotypes, and expectations, these differences increase^71^. In most cultures, women, more than men, are required to attend to the perspectives of others and to take care of others’ interests and desires, which may help to explain the higher interrelation, observed in our study, between their interpersonal attachment insecurities and their cognitive emotion regulation style. In this sense, interpersonal attachment style could be acting in women as a source of additional affective information that, together with the rest of their emotional resources, contributes to the selection of a specific regulatory style^16,72^. This normalized dynamic could be altered in insecure attachment styles; in people with these attachment styles, the associated cognitive biases (e.g., cognitive and emotional distancing in avoidant attachment, or negative evaluation and high emotional reactivity in anxious attachment) would act as a contextual factor, favouring the selection of a higher-risk emotional regulation style, i.e., the *Vulnerable* style.

In sum, our research confirms that women and men display similar cognitive emotion regulation styles and that cognitive strategies considered more adaptive (*Acceptance*, *Positive Refocusing*, *Refocus on Planning*, *Positive Reappraisal*, and *Putting into Perspective*) play a decisive role that predicts their belonging to one or another cognitive emotion regulation style. In turn, although our work does not provide an explanation for some of the observed gender differences, it does highlight some interesting points that may improve our understanding of them. First, although the styles of cognitive emotion regulation were similar in women and men, men apparently presented a wider range of adaptive strategies by including “*Blaming Others*” in the *Protective* style of cognitive emotion regulation strategies. Second, the cognitive emotion regulation styles of men were more independent of their interpersonal affective system than was the case in women, which may be related to the gender differences observed in the socio-cognitive development of women and men in most cultures. The observed gender differences highlight the style of bonding, with the couple as a factor of special relevance in the dynamics of the emotional regulation of women. In this sense, our results suggest the relevance of considering the extent to which toxic and abusive romantic relationships may affect the individual’s cognitive way of dealing with threats or challenges.

## Data Availability

The data of the current study are available from the corresponding author upon reasonable request.
